# PLK4 is a key molecule in the formation of PGCCs and promotes invasion and migration of progeny cells derived from PGCCs

**DOI:** 10.7150/jca.74211

**Published:** 2022-07-18

**Authors:** Fangmei Fu, Lankai Chen, Xiaohui Yang, Linlin Fan, Mingqing Zhang, Shuo Chen, Minying Zheng, Ming Gao, Shiwu Zhang

**Affiliations:** 1 Department of Pathology, Tianjin Union Medical Center, Nankai University, Tianjin, 300121, P.R. China.; 2 Nankai University School of Medicine, Nankai University, Tianjin, 300071, P.R. China.; 3 Graduate School, Tianjin University of Traditional Chinese Medicine, Tianjin, 301617, P.R. China.; 4 Department of Colorectal Surgery, Tianjin Union Medical Center, Tianjin, P.R. China.; 5 Department of Thyroid Surgery, Tianjin Union Medical Center, Tianjin, P.R. China.

**Keywords:** PLK4, CDC25C, bufalin, colorectal cancer, polyploid giant cancer cells, epithelial-mesenchymal transition

## Abstract

**Purpose:** Cancer stem cells (CSCs) are the evil source of tumor metastasis and recurrence. Polyploid giant cancer cells (PGCCs) that exhibit the characteristics of CSCs produced daughter cells via asymmetric division. The molecular mechanisms of daughter cells derived from PGCCs with high migration, invasion, and proliferation abilities in colorectal cancer (CRC) are explored in this paper based on the bioinformatics analysis.

**Materials and Methods:** We characterized the expression of CSC-related genes in CRCs by analyzing the mRNAsi of The Cancer Genome Atlas and survival time. Weighted gene co-expression network analysis was performed to identify the modules of the hub and key genes. The migration, invasion, and proliferation abilities of cells, the expression of epithelial-mesenchymal transition (EMT)-related proteins and polo-like kinase 4 (PLK4) were compared in LoVo and Hct116 cells with and without bufalin treatment. In addition, the expression and subcellular location of cell division cycle 25C (CDC25C) in cells before and after PLK4 knockdown were assessed.

**Results:** Eight hub genes were screened out and positively association with mRNAsi in CRCs based on bioinformatic analysis. Among them, checkpoint Kinase-1 (*CHEK1*), budding uninhibited by benzimidazoles 1 Homolog Beta (*BUB1B*) and *PLK4* were closely associated with the prognosis of CRC patients. Bufalin could induce the formation of PGCCs in LoVo and Hct116 cell lines. PLK4 was overexpressed in PGCCs with progeny cells and progeny cells derived from PGCCs had strong migration and invasion abilities by expressing epithelial-mesenchymal transition (EMT)-related proteins. PLK4 could interact with CDC25C and promote CDC25C phosphorylation which was associated with the formation of PGCCs. Decreasing CDC25C expression in both LoVo and Hct116 PGCCs with progeny cells, while levels of pCDC25C-ser216 and pCDC25C-ser198 were increased in LoVo and decreased in Hct116 PGCCs with progeny cells. pCDC25C-ser216 located in the cytoplasm and pCDC25C-ser198 located in the nucleus in cells after bufalin treatment. Furthermore, expression of CDC25C, pCDC25C-ser216, and pCDC25C-ser198 was downregulated after PLK4 knockdown. Furthermore, the expression level of PLK4 was associated with differentiated degree, and lymph node metastasis in human CRC tissues.

**Conclusion:** PLK4 contributes to the formation of PGCCs by regulating the expression of CDC25C and is associated with the expression and subcellular location of CDC25C, pCDC25C-ser216 and pCDC25C-ser198.

## Introduction

It is reported that by 2030, the number of colorectal cancer (CRC) patients will increase by 60% with 2.2 million additional diagnosed cases 1, 2. Increasing evidence has demonstrated that cancer stem cells (CSCs) were major factors affecting the therapeutic effects of CRCs 3. mRNAsi (the stemness index based on mRNA expression) reflects the gene expression characteristics of stem cells, is a kind of quantization of stem cell characteristics 4. The Cancer Genome Atlas (TCGA) project provides comprehensive data on key genomic sequences from major cancer types, representing a significant advance in cancer genomics 5. Through analyzing the RNA-sequencing data downloaded from TCGA database and based on mRNAsi, we eventually screened out eight key differentially expressed genes (DEGs), namely polo like kinase 4 (*PLK4*), DNA replication helicase 2 (*DNA2*), checkpoint Kinase-1 (*CHEK1*), kinesin family member 23 (*KIF23*), budding uninhibited by benzimidazoles 1 Homolog Beta (*BUB1B*), minichromosome maintenance 10 (*MCM10*), threonine tyrosine kinase (*TTK*) and Budding uninhibited by benzimidazoles 1 (*BUB1*), all of which were significantly highly expressed in colorectal CSCs. Among these genes, three genes (*BUB1B, CHEK1, PLK4*) were closely associated with survival time of CRC patients.

Polyploid giant cancer cells (PGCCs) are a special type of CSCs that differ from diploid tumor cells in their size and exhibit chemo-radiotherapy resistance and invasion and migration abilities 6, 7. We previously have demonstrated that nuclear translocation failure of CDC25C resulted in G2/M arrest, promoting the formation of PGCCs 8. PLK4, a serine/threonine kinase, is paramount to centriole duplication during mitosis 9-12. A study showed that PLK4 interacted with CDC25C and phosphorylated CDC25C 13. In addition, another study indicated that PLK4 could promote epithelial-mesenchymal transition (EMT) to accelerate the migration and invasion abilities of CRC cells through the Wnt/β-catenin signaling pathway 14.

Bufalin, a famous traditional Chinese medicine, is usually used to treat malignant tumors, including CRC, liver cancer, lung cancer, acute promyelocytic leukemia 15. Bufalin could kill hepatoma cells by arresting the cell cycle in the G2/M phase 16, 17. Treatment with bufalin increased the expression of CHEK1 and decreased the expression of CDC25C, pCDC25C-Ser198, CDK1, pCDK1-Thr161, cyclin A, and cyclin B 17. In our study, bufalin was used to induce the formation of PGCCs, and PLK4 was selected to verify in the PGCCs with their progeny cells. The molecular mechanism of PLK4 in PGCC formation and its role in promoting the migration and invasion of progeny cells derived from PGCCs were explored in this study.

## Materials and methods

### 1. Download and disposal data

Four hundred and seventy six cases of clinical data from human CRC patients were downloaded. The criteria for the inclusion of patients downloaded from the database included: (1) patients do not receive chemotherapy and/or radiotherapy prior to the radical operation; (2) patients did not accompany any other tumors; (3) patients' age was at least 18 years. The criteria for exclusion included: (1) The clinicopathologic data of patients was incomplete; (2) patients' age was less than 18 years. According to the inclusion and exclusion criteria, 419 cases of CRCs were finally involved in this study. RNA-sequencing data of 42 normal colon samples and 488 CRC samples were downloaded from TCGA database. The data are the most recently collected till March 9, 2021. First, by using the Perl language script, we merged the RNA-sequencing data obtained from 530 tissues into a matrix file. Then, we changed the Ensembl IDs to gene symbols using the Ensembl database.

### 2. Clinical characteristic analysis and screening for DEGs

The prognostic value of mRNAsi in 419 cases of human CRC and the relationship of mRNAsi with CRC stages and CRC grades using the survival package and beeswarm package in R software were predicted, respectively. Furthermore the limma package in R was used to screen DEGs between normal tissues and CRC tissues. False discovery rate<0.05, and |log2 FC|>1 were considered as filtered conditions. Finally, the limma and pheatmap packages in R were performed to visualize the DEGs as volcano plots and heatmaps.

### 3. Weighted gene co-expression network analysis (WGCNA)

WGCNA is a system biology method that can be used to describe gene correlation patterns in microarray samples 18. The WGCNA package in R can be used to perform WGCNA analyses 18. The expression levels of screened DEGs were analyzed and the value was calculated to recognize the key module. Gene significance (GS) was used to calculate the association between genes and samples. The average GS of all genes in the module was used to measure the significance of the module, and observed the relationship among different modules with sample traits. The module with the highest correlation was chosen as the key module. After that, GS and module membership (MM) were calculated. We set cor. gene MM>0.8 and cor. gene GS>0.5 as the threshold for screening key genes in the modules.

### 4. Key gene identification and survival curve

Eight hub genes were identified after WGCNA analysis. The TCGAportal online tool (http://tumorsurvival.org/) was used to plot the survival curves based on the expression of eight key genes.

### 5. Cell lines and culture

The normal colon epithelial cell line ncm460, and colon cancer cell lines LoVo and Hct116 were purchased from the American Type Culture Collection (ATCC). These two cell lines were cultured in RPMI-1640 medium (Thermo Fisher Scientific). Cells were cultured at 37^◦^C in a 5%CO_2_ incubator.

### 6. The formation of PGCCs induced by Bufalin

Bufalin (800 nM and 1600 nM; Chenguang, Baoji City, China) was added to LoVo and Hct116 for 48h, respectively. After bufalin treatment, a large proportion of cells died. Only a few giant cells (PGCCs) survived in the culture flask. After 14 days of bufalin treatment, PGCCs produced progeny cells via asymmetric division. After bufalin treatment for three times, 20%-30% of the population in the flask were PGCCs, 70%-80% cells were progeny cells with small size.

### 7. Real-time PCR (RT-PCR) Analysis

Total RNA from LoVo and Hct116 cells and PGCCs with progeny cells was isolated using TRizol reagent (Invitrogen, USA) and reversely transcripted into cDNA according to the manufacturer's instructions (Novcare Biotech, China). For Taqman PCR, each reaction contained 2 μL of cDNA diluted in nuclease free water, 10 μL of Universal PCR Master Mix (CWBIO, 0957), 0.5 μL of 10 μM forward primer and 0.5 μL of 10 μM reverse primer with nuclease-free water were added to a final volume of 20 μL. Amplification and detection were performed using the ABI Prism 7500 sequencer. The detection system (PE Applied Biosystems) and the thermal cycle program used in this study were: 10 min at 95°C, followed by 60 cycles of 15 s denaturation at 95°C and 1 min annealing at 60°C. The level of PLK4 expression was normalized to that of glyceraldehyde 3-phosphate dehydrogenase (GAPDH). The PLK4 primer sequences were 5'-CCT TAT CAC CTC CTC CTT C-3' and 5'-CCA AGT CCT TCA TTT GTA ACC-3'. The GAPDH primer sequences were 5'-ACCACAGTCCATGCCATCAC-3′ and 5′-TCCACCACCCTG TTGCTGTA-3′.

### 8. Total protein extraction and cytoplasmic and nuclear protein isolation assay

Cells were lysed in radioimmunoprecipitation assay (RIPA) buffer on ice for 0.5 h and then centrifuged at 14000 rpm for 0.5 h at 4 °C. The reagents used for cytoplasmic and nuclear protein isolation were obtained from Beyotime Biotechnology (Shanghai, China). First, 200 µl reagent A (supplemented freshly with PMSF 2 µl) was added to 20 µl cell pellets and mixed at 4℃ for half an hour. Reagent B (10 µl; reagent A: reagent B=20:1) was then added and the lysates were centrifuged at 14,000 rpm for 0.5 h at 4℃. The supernatant was then moved into a cold new centrifugal tube, the pellet was rinsed 3 times with PBS, and 50 µl nuclear protein extraction reagent (containing PMSF) was added to the pellet. Pellet was dissolved with high-speed eddy at 4℃ for 0.5 h and then centrifuged at 14000 rpm for 0.5 h at 4℃. The resulting liquid supernatant was moved to a new cold centrifugal tube.

### 9. Western blots (WB) analysis

The cells were lysed as described above. For graft tissue, 20 mg of tissue was weighed, 200μl RIPA buffer was added, and the tissues were then lysed on ice. The remaining steps were the same as those used for cell lysis. Protein samples were loaded on 8% or 10% sodium dodecyl sulfate-polyacrylamide gels and then separated by electrophoresis. After that, the separated proteins were transferred onto PVDF membranes (Millipore, USA). The membranes containing proteins were soaked in 5% defatted milk (BD, USA) up to 2 h at room temperature for blocking. The membranes were then incubated with primary antibodies (Supplementary Table 1) at 4 °C last at least overnight. Next day, secondary antibodies were used to react with primary antibodies through incubating for 2 h at room temperature. Protein expression was evaluated using a ChemiDoc imaging system (Bio-Rad, USA).

### 10. Immunocytochemical (ICC) staining

Cells were cultured on coverslips embedded in the 6-well plate for one or two days, and fixed using cold anhydrous methanol for 1 h until the confluency reached 90%. Next, endogenous peroxidase activity was blocked by endogenous peroxidase inhibitor (Zhongshan Inc., Beijing, China) followed with goat serum (Zhongshan Inc., Beijing, China) at room temperature for 15 min, respectively. Then, samples were incubated with primary antibodies (Supplementary Table 1) at 4°C overnight, and next day incubated with a secondary antibody for 1 h. Horseradish peroxidase-labeled streptomycin (Zhongshan Inc.) was then added to cells for 30 min. Diaminobenzidine was used to stain the proteins, and hematoxylin was used for counterstaining.

### 11. Wound healing assay

Wound healing assay was performed to assess the migration ability in different groups. Cells were seeded in 6-well plates for growing. When the confluency reached 90%, four horizontal lines were scratched at the bottom by a sterile pipette cusp. The old medium was discarded and sterile PBS was used to slowly wash the cells followed by adding new serum free RPMI 1640 medium. Cells were then photographed at 0 and 24 h, respectively. The scratched area was measured using ImageJ software.

### 12. Cell migration and invasion assay

The transwell migration assay was used by using chambers (Corning) and 24-well plates. Upper chamber with 200 µL serum free medium contained 1×10^5^ cells while 600 µL medium with 10% serum was added to the lower chamber. Twenty-four hours later, cells were fixed in cold anhydrous methanol for 30 min and subsequently stained by 0.1% crystal violet for 30 min. Five fields were used to observe the cells that had penetrated the membrane. The process of transwell invasion assay was generally the same, except to use chambers precoated with matrigel (Corning) instead of those without matrigel.

### 13. Plate colony formation assay

The proliferative ability of cells was assessed by using a plate colony formation assay. Each group was cultured with 30, 60, or 120 cells in 12-well plates for 2 weeks at 37 ℃ with 5% CO2. Fifteen days later, cell colonies were fixed in cold anhydrous methanol for 30 min and subsequently stained by 0.1% crystal violet for 30 min. The number of cell colonies was counted such that a single colony must contain at least 50 cells.

### 14. Cell counting kit-8 (CCK8) assay

CFI-400945 can be used as a selective and orally active inhibitor of PLK4 19, 20. Viability of bufalin-treated LoVo and Hct116 cells with CFI-400945 treatment was assessed by CCK8 assay. Bufalin-treated LoVo and Hct116 cells (4×10^3^ cells) were incubated by CFI-400945(MCE, USA) with progressive increase concentration (0, 4 μM, 8 μM, 16 μM, or 32 μM) and different time (12 h, 24 h, 36 h, or 48 h), respectively. CCK8 working liquid (10%; Dojndo, Japan) was used to incubate for 1 h. An enzyme-labelled instrument was used to measure the absorbance of each well and determine the appropriate reaction time and concentration of CFI-400945 (LoVo PGCCs with progeny cells: 8 µM for 36 h; Hct116 PGCCs with progeny cells: 8 µM for 48 h).

### 15. Transient small interfering RNA transfection

The PLK4 siRNA was purchased from Gene-Pharma (Shanghai, China) and transfected into the cells using Lipofectamine RNAiMAX (Invitrogen). Sequence information about siRNA used is listed in Supplementary Table 2.

### 16. Immunohistochemical (IHC) staining

Xylene was used to dewax the paraffin-embedded tissue, which was then rehydrated with gradient alcohol solutions. To restore antigens, these sections were immersed in boiled citrate buffer solution (Zhongshan Inc.) for 3 minutes. Next, endogenous peroxidase activity was blocked by endogenous peroxidase inhibitor followed with goat serum blocking the nonspecific background. Then these sections were incubated with primary antibody against PLK4 (Supplementary Table 1) at 4°C overnight. The following steps were consistent with ICC staining.

### 17. Scoring of IHC staining

Staining was considered positive when the cytoplasm and nucleus appeared as brown-yellow. The standards for IHC evaluation can be found in our previous article 21. Simply, the stain index is used for the scoring of IHC staining. The staining intensity was assessed as follows: 0, negative (no staining); 1, weakly positive (faint yellow staining); 2, moderately positive (brownish-yellow staining); and 3, strongly positive (brown staining). The number of positive cells was visually evaluated as follows: 0 (negative), <5% positive cells; 1 (weak), 6%-25% positive cells; 2 (moderate), 26%-50% positive cells; 3 (above moderate), 51%-75%; and 4 (strong), >76% positive cells. The stain index for each section is the sum of staining intensity and positive cell scores.

### 18. Animal experiments

Tumor xenografts were performed by inoculation of LoVo and Hct116 cells treated with or without bufalin. Twenty BALB/cNU/NU nude mice (7-week-old) were brought from Beijing Weitonglihua Co., Ltd. and were then randomly divided into four groups: LoVo control cells, bufalin-treated LoVo cells, Hct116 control cells, and bufalin-treated Hct116 cells (five mice per group). The flanks of the mice were injected with 100 µL of PBS containing 1×10^6^ cells. Thirteen days after inoculation with LoVo cells and five days after inoculation with Hct116 cells, the tumor mass was measured every two days. Tumor volume (mm^3^)=(length×width^2^)/2. Forty-three days after inoculation with LoVo cells and seventeen days after inoculation with Hct116 cells, mice were sacrificed and their tumor tissues were isolated for further analysis. The animal experiments were approved by the Animal Care and Committee of the Tianjin Union Medicine Center.

### 19. Human CRC samples

Two hundred and three human paraffin-embedded CRC tissue samples from 158 CRC patients were collected from the department of pathology in our institute. Based on the pathological results, these tissues were divided into four groups: Group I: 46 cases of well-differentiated CRCs; group Ⅱ: 53 cases of moderately differentiated CRCs; group III: 48 cases of poorly differentiated CRCs; group IV: 56 cases of lymph node metastatic foci. The experiments were approved by the hospital review board of Tianjin Union Medical Center and the confidentiality of patient information was maintained.

### 20. Statistical analysis

SPSS (version 26.0) was used to perform statistical analyses. An independent samples T test was used to compare protein expression levels and xenograft volumes in mice. The Kruskal-Wallis assay was used to compare PLK4 expression in human CRCs. All column diagrams are expressed as mean±SD. *P*<0.05 was considered as statistically significant.

## Results

### 1. Stemness indices and clinical significance in CRCs

**Figure 1:**
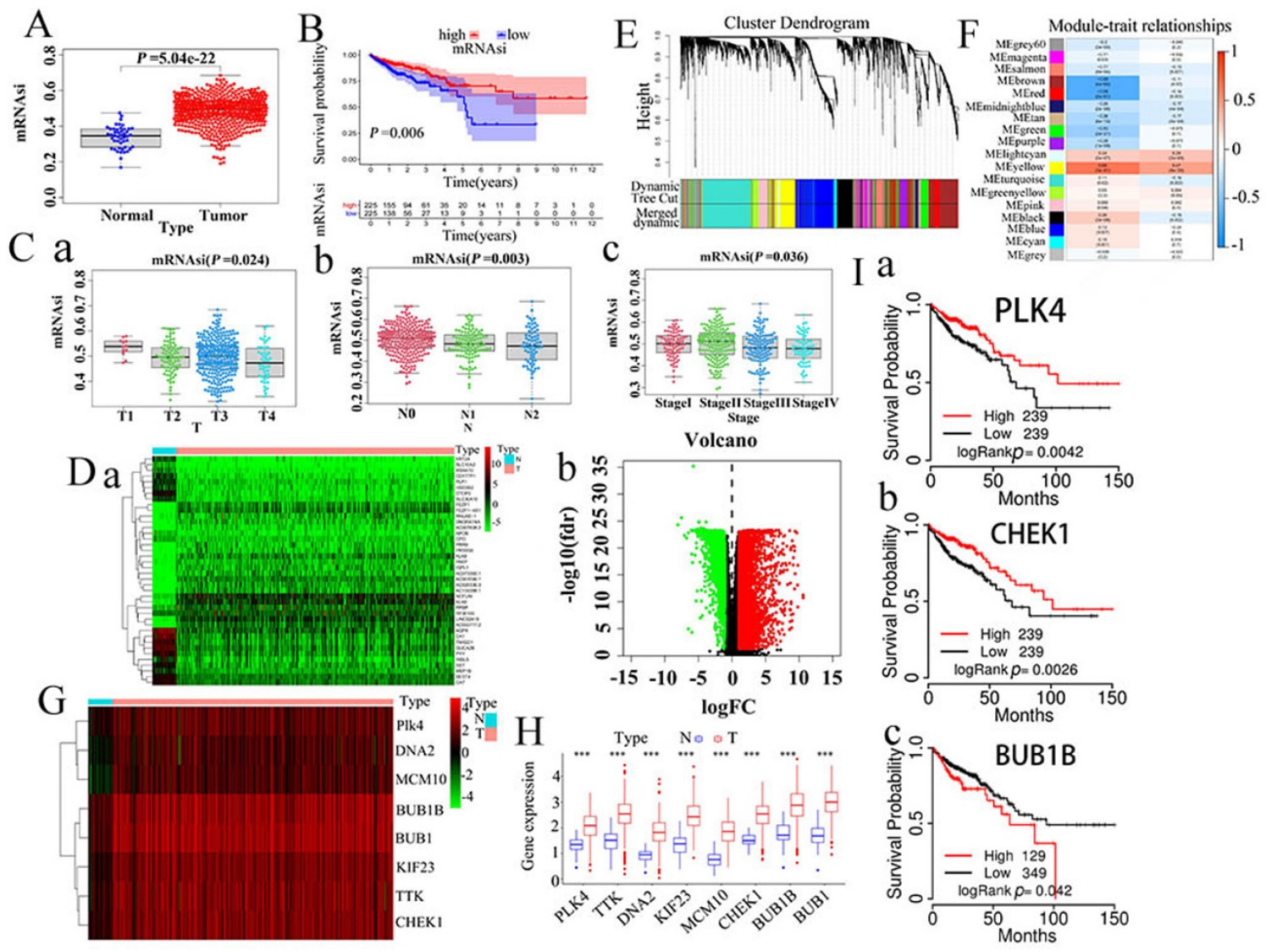
A. Comparision of mRNAsi in normal and CRC tissues. B. Kaplan-Meier survivorship curve of mRNAsi in CRC. C (a-c). Different mRNAsi in different clinical stages of CRCs. D. Differentially expressed genes showed in a. heatmap and b. Volcano plots (green represents downregulated genes while red represents upregulated genes). E. Clustering dendrograms of similar DEGs were showed based on WGCNA analysis. F. Correlation between gene modules and mRNAsi. G. Heatmap of the eight hub genes. H. Comparisons of the expression of the screened eight key genes in CRC tissues and normal intestinal tissues based on TCGA database. I. Survival curve with regard to expression of the three screened genes (a.*PLK4*, b. *CHEKI*, c. *BUB1B*) by using TCGA portal online tool. DEGs, differently expressed genes. CRC, colorectal cancer. WGCNA, weighted gene co-expression network analysis; TCGA, The Cancer Genome Atlas.

mRNAsi can be considered as a quantitative characterization of CSCs, acting as an indicator performed to describe the degree of similarity between tumor cells and stem cells. In our study, mRNAsi appeared a significant difference between normal and CRC tissues, and CRC tissues have higher mRNAsi than normal tissues (*P*<0.001) (Fig. 1A). Survival analysis showed that mRNAsi was closely associated with survival time of cancer patients (Fig. 1B). According to the TNM staging, the mRNAsi scores decreased gradually from T1 to T4 (Fig.1 C-a) and from N0 to N2 (Fig. 1C-b). In CRC patients with stage Ⅳ, the tumor cells had the lowest mRNAsi score compared with it in CRC patients with stage Ⅰ, Ⅱ, and Ⅲ (Fig. 1C-c).

### 2. Screening of DEGs

Because mRNAsi levels were significantly different between normal and tumor tissues, we filtered DEGs using data cleansing. Subsequently, normal and CRC samples were normalized. From this analysis, we eventually screened 6477 DEGs, of which 4414 were upregulated while 2063 were downregulated, as shown by heatmaps (Fig. 1Da) and volcano plots (Fig. 1Db).

### 3. WGCNA: screening of the most significant modules and genes

WGCNA can be used to identify biologically significant gene modules and construct gene co-expression networks, thus making it possible to gain a better understanding of the genes associated with stemness in CRCs. In this study, 17 modules were obtained for further study (Fig. 1EF). Among them, the yellow module had a correlation value close to 0.7, suggesting that it had the closest correlation with mRNAsi. Therefore, the yellow module was selected for further study. In addition, the black, blue, and yellow modules also displayed high correlations with mRNAsi (Fig. S1A-abc). Finally, we screened out eight key genes: *PLK4, BUB1B, KIF23, CHEK1, BUB1, MCM10, DNA2*, and *TTK.* The specific expression values of the eight key genes were visualized using heat maps (Fig. 1G) and boxmaps (Fig. 1H), and the results showed that these eight key genes were significantly overexpressed in CRCs (Fig. 1H).

### 4. Survival curve with regard to expression of the eight key genes

TCGAportal online tool was used to investigate the survival of CRC patients with respect to the expression of the eight key genes, and eventually three genes (*BUB1B,CHEK1, PLK4*) showed an evident correlation with CRC patients in terms of survival (Fig. 1I, Fig. S1B-abcde).

### 5. Bufalin induces the formation of PGCCs in LoVo and Hct116

**Figure 2:**
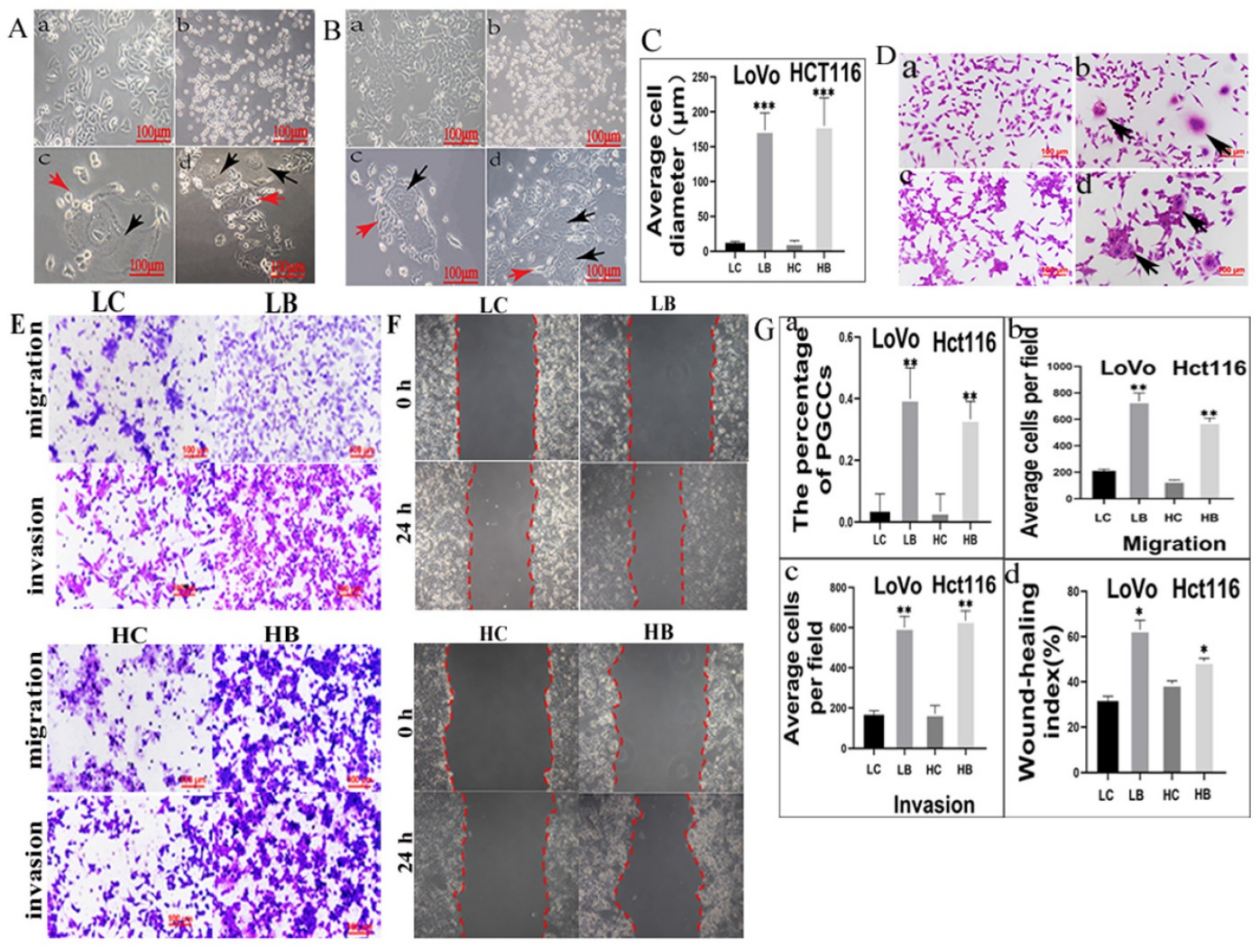
A. LoVo cells with or without bufalin treatment (100×). (a) LoVo cells with no bufalin treatment. (b) LoVo cells treated with 800 nM bufalin for 48 h. (c) Individual PGCC surviving after 800 nM bufalin treatment. (d) Increasing number of PGCCs and their budding cells. Red arrow points to progeny cells and black arrow points to PGCC. B. Hct116 cells with or without bufalin treatment (100×). (a) Hct116 cells with no bufalin treatment. (b) Hct116 cells treated with 1600 nM bufalin for 48 h. (c) Individual PGCC surviving after 800 nM bufalin treatment. (d) Increasing number of PGCCs and budding cells. Red arrow points to progeny cells and black arrow points to PGCC. C. The difference in average cell diameter before and after bufalin treatment is statistical significant (***P<0.001). D. H&E staining of LoVo and Hct116 cells with and without bufalin (200×). (a) LoVo cells with no bufalin treatment, (b) Several LoVo PGCCs (Black arrows head) appeared after bufalin treatment, (c) Hct116 cells with no bufalin treatment, (d) Several Hct116 PGCCs (Black arrows head) appeared after bufalin treatment. E. Comparison of migration and invasion abilities in cells before and after bufalin treatment using transwell assay (200×). F. Comparison the migration ability in cells before and after bufalin treatment using wound-healing assay. G. Statistical diagram. a. Comparison of the percentage of PGCCs in LoVo and Hct116 cells before and after bufalin treatment (**P<0.01). Column diagram of transwell migration (b) and invasion assay(c) wound-healing assay(d) in LoVo and Hct116 cells before and after bufalin treatment(*P<0.05, **P<0.01). LC, LoVo control cells. HC, Hct116 control cells. LB, LoVo cells treated with bufalin. HB, Hct116 cells treated with bufalin.

Few of PGCCs could be observed in LoVo and Hct116 control cells. After treatment with 800 nM and 1600 nM bufalin in LoVo and Hct116 cells (Fig. 2A-a and 2B-a), respectively, for 48 h, the majority of the small-sized tumor cells died (Fig. 2A-b and 2B-b) and only the PGCCs survived from the treatment. The morphological characteristics of PGCCs with large volume (Fig. 2A-c and 2B-c) were completely different from those of small-sized cells. After about two weeks, PGCCs recovered from bufalin treatment and began to produce progeny cells via asymmetric division (Fig. 2A-d, 2B-d). The differences of the average cell diameter between small-sized tumor cells and PGCCs were statistically significant (Fig. 2C). Results of H&E staining results also showed that the number of PGCCs were increased after bufalin treatment (Fig. 2D-a-d), and the number of PGCCs in LoVo and Hct116 treated with and without bufalin had statistically significance (Fig. 2G-a).

### 6. Migration, invasion, and proliferation abilities of progeny cells generated from PGCCs

**Figure 3:**
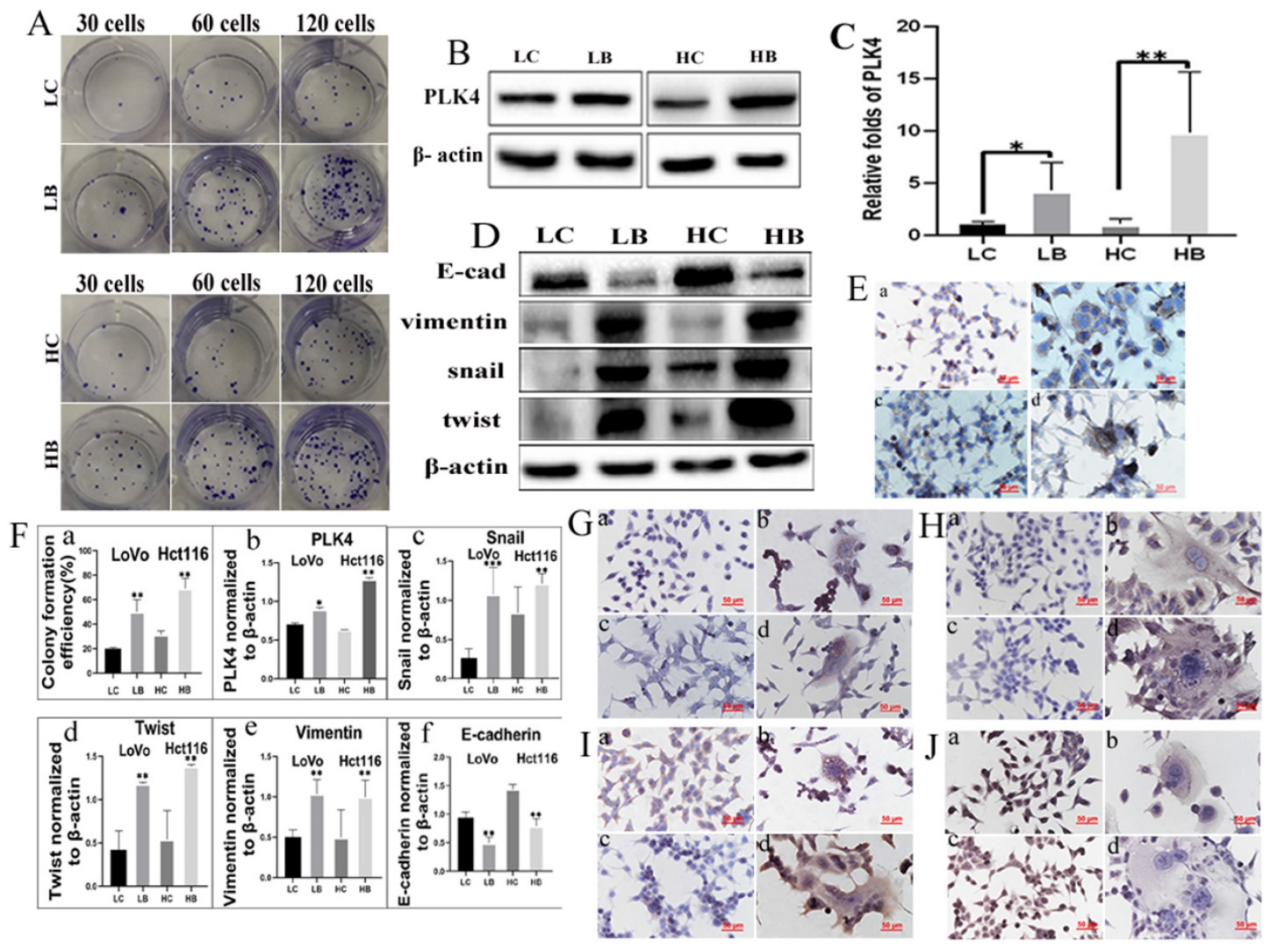
A. Comparison of the number of cell colonies after 30, 60, and 120 LoVo and Hct116 cells were incubated in the 12-well plates, respectively. Cells were either untreated or treated with bufalin. B. Result of WB showed PLK4 expression level of LoVo and Hct116 cells with and without bufalin treatment. C. RT-PCR analysis of mRNA expression of PLK4 in LoVo and Hct116 cells before and after bufalin treatment. D. WB analysis of E-cadherin, twist, vimentin and snail expression in LoVo and Hct116 cells before and after bufalin treatment. E. ICC staining of PLK4 in LoVo and Hct116 cells before and after bufalin treatment (200×). (a) LoVo control cells, (b) PLK4 in LoVo cells after bufalin treatment, (c) Hct116 control cells, (d) PLK4 in Hct116 cells after bufalin treatment. F. Column diagram showed that differences of colony formation assay, PLK4, snail, twist, vimentin and E-cadherin in LoVo and Hct116 cells before and after bufalin treatment. a. colony formation assay (**P<0.01). b. PLK4 expression (*P<0.05, **P<0.01). c. snail expression (**P < 0.01, ***P < 0.001). d. twist expression (**P < 0.01). e. vimentin expression (**P < 0.01). f. E-cadherin expression (**P < 0.01). G-J. ICC staining of snail (G), twist (H), vimentin (I) and E-cadherin (J) in LoVo control cells (a), LoVo cells after bufalin treatment (b), Hct116 control cells (c), Hct116 cells after bufalin treatment (d). (200×). LC, LoVo control cells. HC, Hct116 control cells. LB, LoVo cells treated with bufalin. HB, Hct116 cells treated with bufalin. WB, western blot analysis. ICC, Immunohistochemical.

We have verified that bufalin could produce PGCCs and their progeny cells, then cell functional experiments were used to detect the migration, invasion, and proliferation abilities of progeny cells generated from PGCCs. The results of transwell assay illustrated that cells underlying bufalin treatment had stronger migration and invasion (Fig. 2E) abilities than cells without bufalin treatment. Moreover, results of wound-healing assay also confirmed that the migration ability of cells after bufalin treatment was increased in comparison with those cells without bufalin treatment (Fig. 2F), and the differences in the migration and invasion abilities of cells with and without bufalin treatment were statistically significant (Fig. 2G-bcd). The proliferative ability of these two cell lines with and without bufalin treatment was observed using the plate colony formation assay (Fig. 3A), LoVo and Hct116 cells treated with bufalin exhibited stronger proliferative ability compared with the control cells, and the differences had statistically significant (Fig. 3F-a). These results indicated that progeny cells derived from PGCCs owned stronger migration, invasion, and proliferative abilities.

### 7. PLK4 and EMT-related proteins are expressed in PGCCs with progeny cells

The expression of PLK4 was increased in colon cancer cell lines LoVo and Hct116 cells compared with normal colon epithelial cell line ncm460 (Fig. S1C) and the differences were statistically significant (Fig. S1D). The expression of PLK4 was increased in PGCCs with progeny cells from bufalin treatment compared with the control cells (Fig. 3B), and the difference was statistically significant (Fig. 3F-b). mRNA level expression of PLK4 in PGCCs with progeny cells was also detected by RT-PCR and the result showed that mRNA expression level of PLK4 was increased in PGCCs with progeny cells compared with the cells without bufalin treatment, and the difference was statistically significant (Fig. 3C). Results of WB showed that expression of EMT-related proteins including snail, twist, and vimentin were elevated, while E-cadherin expression was downregulated in LoVo and Hct116 PGCCs with progeny cells in contrast with those control cells without bufalin treatment (Fig. 3D), and the differences were statistically significant (Fig. 3F-cdef). Results of ICC staining showed that PLK4 (Fig. 3E), snail (Fig. 3G), twist (Fig. 3H), vimentin (Fig. 3I) were significantly overexpressed, while E-cadherin expression (Fig. 3J) was decreased in cells with bufalin treatment compared with that in cells without bufalin treatment.

### 8. Inhibiting expression of PLK4 decreased the invasion and migration abilities of progeny cells derived from PGCCs after bufalin treatment

PLK4 expression is associated with the invasion and migration abilities of progeny cells generated from PGCCs induced by bufalin. Functional experiments were performed to observe the invasion, migration and proliferative ability of progeny cells derived from PGCCs after inhibiting the expression of PLK4.

**Figure 4:**
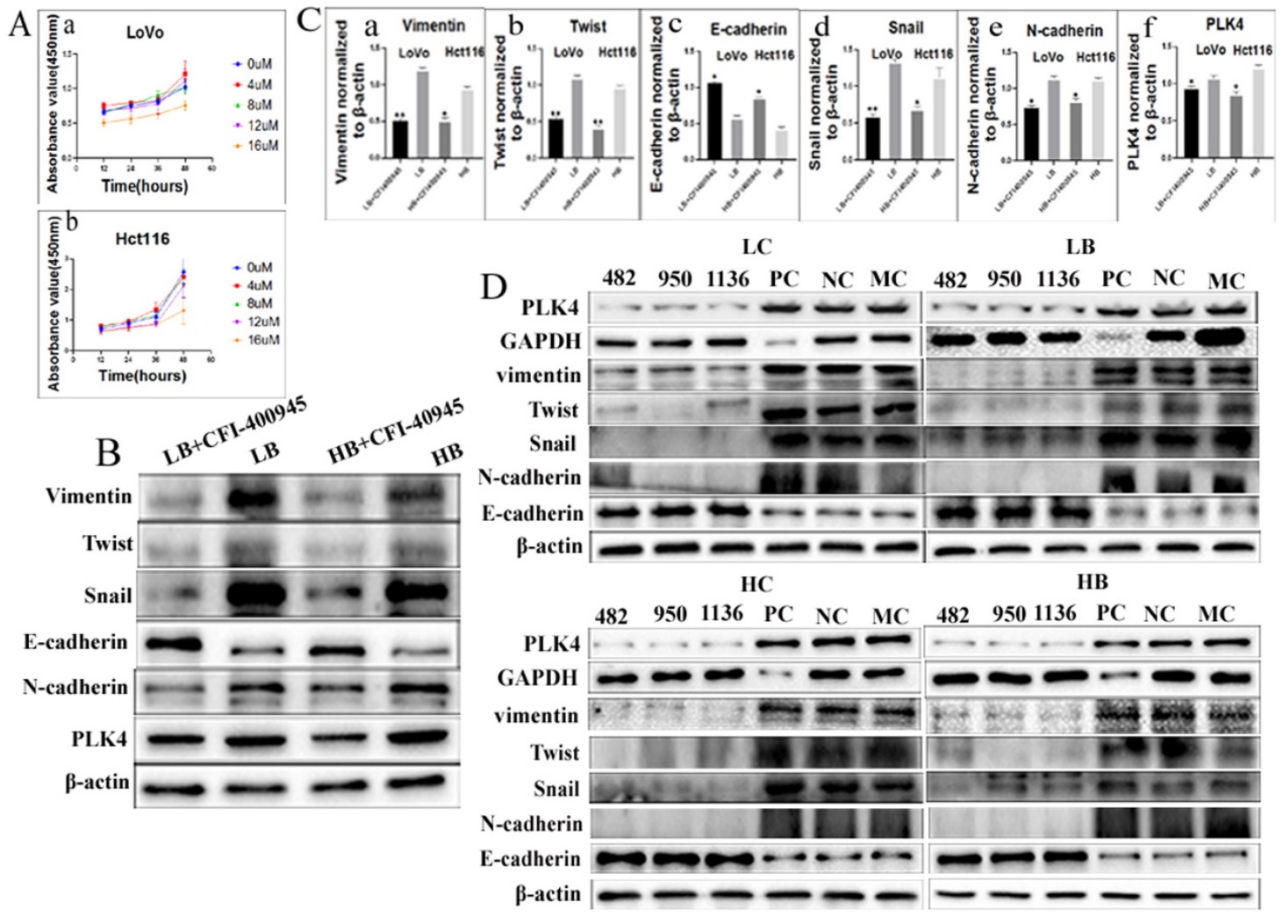
A. The influence of different concentrations of CFI-400945 on the cell viability of (a) LoVo treatment cells and (b) Hct116 treatment cells, was detected by CCK8 assay. B. WB analysis of PLK4, Vimentin, Twist, Snail, N-cadherin, and E-cadherin expression levels in LoVo and Hct116 cells treated with bufalin after 8 µM CFI-400945 treatment for 48 h and 36 h, respectively. C. Difference in the gray value of the expression of Vimentin (a), Twist (b), E-cadherin (c), Snail (d), N-cadherin (e), PLK4 (f) in LoVo cells and Hct116 cells treated with bufalin after 8 µM CFI-400945 treatment for 48 h and 36 h, respectively (**P < 0.01,*P < 0.05). D. WB analysis of PLK4, Vimentin, Twist, Snail, N-cadherin and E-cadherin expression after siRNA-PLK4 transfection into LoVo and Hct116 cells. LC, LoVo control cells. HC, Hct116 control cells. LB, LoVo cells treated with bufalin. HB, Hct116 cells treated with bufalin. MC, Mock control. PC, siRNA GAPDH. NC, negative control. WB, Western blot analysis.

**Figure 5:**
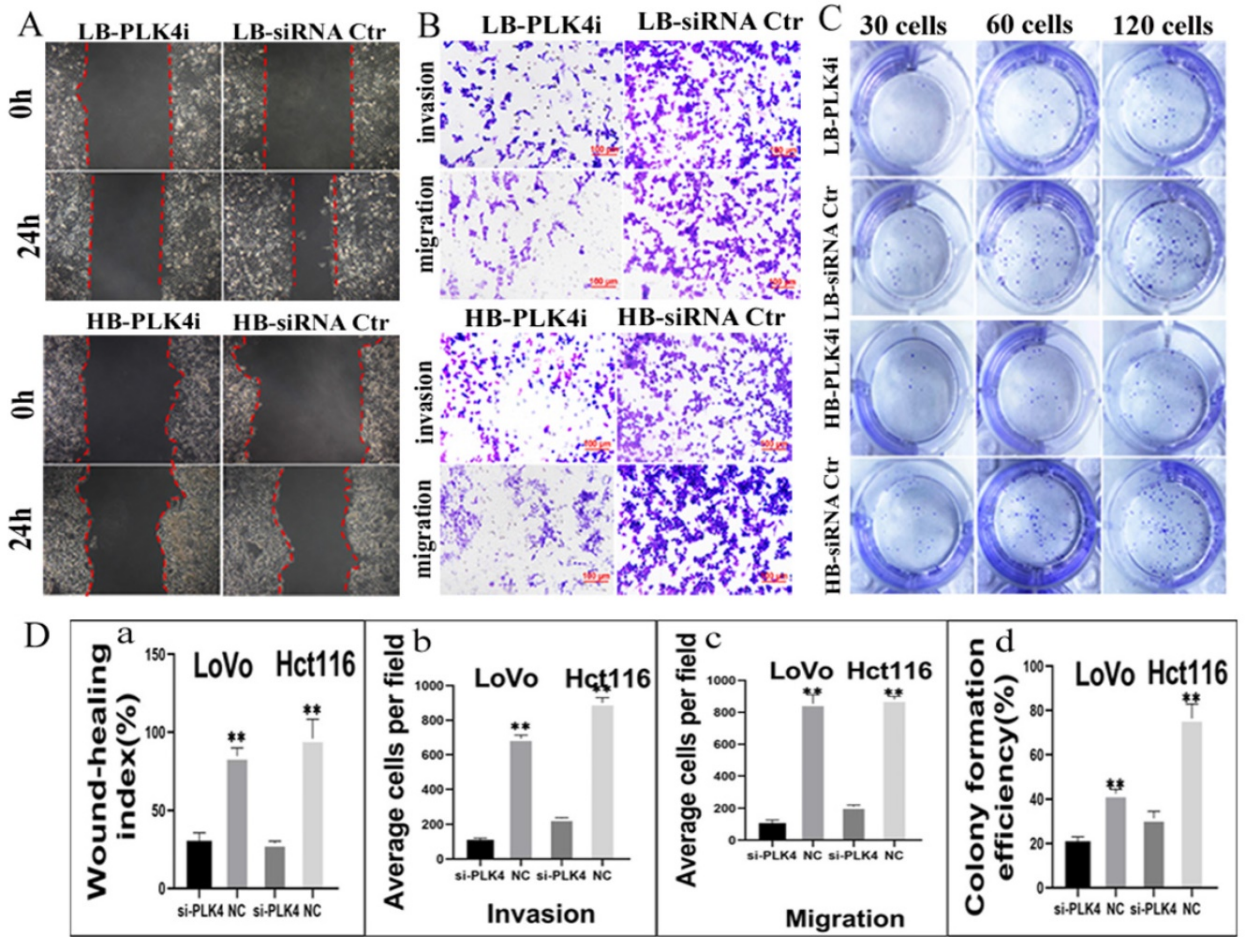
A. Comparison of the migration rate of bufalin-treated LoVo and Hct116 cells after PLK4 knockdown using wound-healing assay. B. Comparison of migration and invasion abilities of bufalin-treated LoVo and Hct116 cells after PLK4 knockdown using transwell assay (200×). C. Comparison of the number of cell colonies after 30, 60, and 120 LoVo and Hct116 bufalin-treated cells with PLK4 knocked down were seeded in 12-well plates. D. Statistical chart of functional experiment. a. Statistical histogram of wound-healing assay after PLK4 knocked down (**P<0.01). b. Statistical histogram of transwell invasion assay in bufalin-treated LoVo and Hct116 cells after PLK4 knocked down (**P < 0.01). c. Statistical histogram of transwell migration assay in bufalin-treated LoVo and Hct116 cells after PLK4 knocked down (**P<0.01). d. Statistical histogram of cell cloning assay in bufalin-treated LoVo and Hct116 cells after PLK4 knocked down (**P < 0.01). LC, LoVo control cells. HC, Hct116 control cells. LB, LoVo cells treated with bufalin. HB, Hct116 cells treated with bufalin. PLKi, PLK4 siRNA.

CFI-400945 was a selective inhibitor of PLK4 and employed to suppress the expression of PLK4 20. Results of CCK8 confirmed that the optimum concentration and time of LoVo was 8 µM for 36 h and Hct116 was 8 µM for 48 h (Fig. 4Aa-b), respectively. Both transwell migration assay and wound-healing assay displayed that the migration ability of cells treated with CFI-400945 were lower than that of PGCCs and progeny cells without CFI-400945 treatment (Figs. S2AB). Transwell invasion assay demonstrated that the invasion ability of PGCCs with progeny cells treated with CFI-400945 was also descended in comparison with those without treatment of CFI-400945 (Fig. S2AB). The proliferative ability of cells treated with CFI-400945 was also reduced as shown by plate colony formation assay (Fig. S2C). The differences of cell functional experiments in PGCCs with progeny cells with and without CFI-400945 treatment were all statistically significant (Fig. S2Dabcd). In addition, transient transfection targeting PLK4 was used to inhibit the expression of PLK4. The migration, invasion and proliferative abilities of PGCCs with progeny cells were all decreased after PLK4 knockdown (Fig. 5ABC), and the difference were all statistically significant (Fig. 5Dabcd). These cell functional experiments revealed that the invasion, migration and proliferative abilities of progeny cells derived from PGCCs were decreased after inhibition of PLK4 expression by using small molecule inhibitor CFI-400945 or PLK4 siRNA.

### 9. Expression of EMT-related proteins after inhibiting PLK4 expression

In order to explore the molecular mechanism by which PLK4 promoting the invasion, migration and proliferative abilities of progeny cells, the expression of EMT-related proteins were detected in cells after inhibiting PLK4 expression by using small molecule inhibitor CFI-400945. Results of WB showed that the expression of snail, N-cadherin, vimentin, twist were declined and E-cadherin was increased in LoVo and Hct116 cells (Fig. 4B). The differences of PLK4 and EMT-related proteins expression in LoVo and Hct116 cells before and after CFI-400945 treatment were statistically significant (Fig. 4Cabcdef). Further the expression levels of EMT-related proteins were detected in cells after PLK4 knockdown. Results of WB confirmed that snail, N-cadherin, vimentin, twist and E-cadherin expression in LoVo and Hct116 after PLK4 knockdown were consistent with those in cells after CFI-400945 treatment (Fig. 4D). Evidences from these data suggested that PLK4 promoting the invasion, migration, and proliferative abilities of progeny cells derived from PGCCs might be associated with the expression of EMT-related proteins.

### 10. Expression of CDC25C, pCDC25C-ser216, and pCDC25C-ser198 in cells after bufalin treatment

We have demonstrated that CDC25C decreased expression in the cell nucleus and associated with formation of PGCCs in HEY, BT-549, SKOV3 and MDA-MB-231 cells 8. In this study, the expression of CDC25C, pCDC25C-ser216 and pCDC25C-ser198 were detected in LoVo and Hct116 cells with and without bufalin treatment.

**Figure 6:**
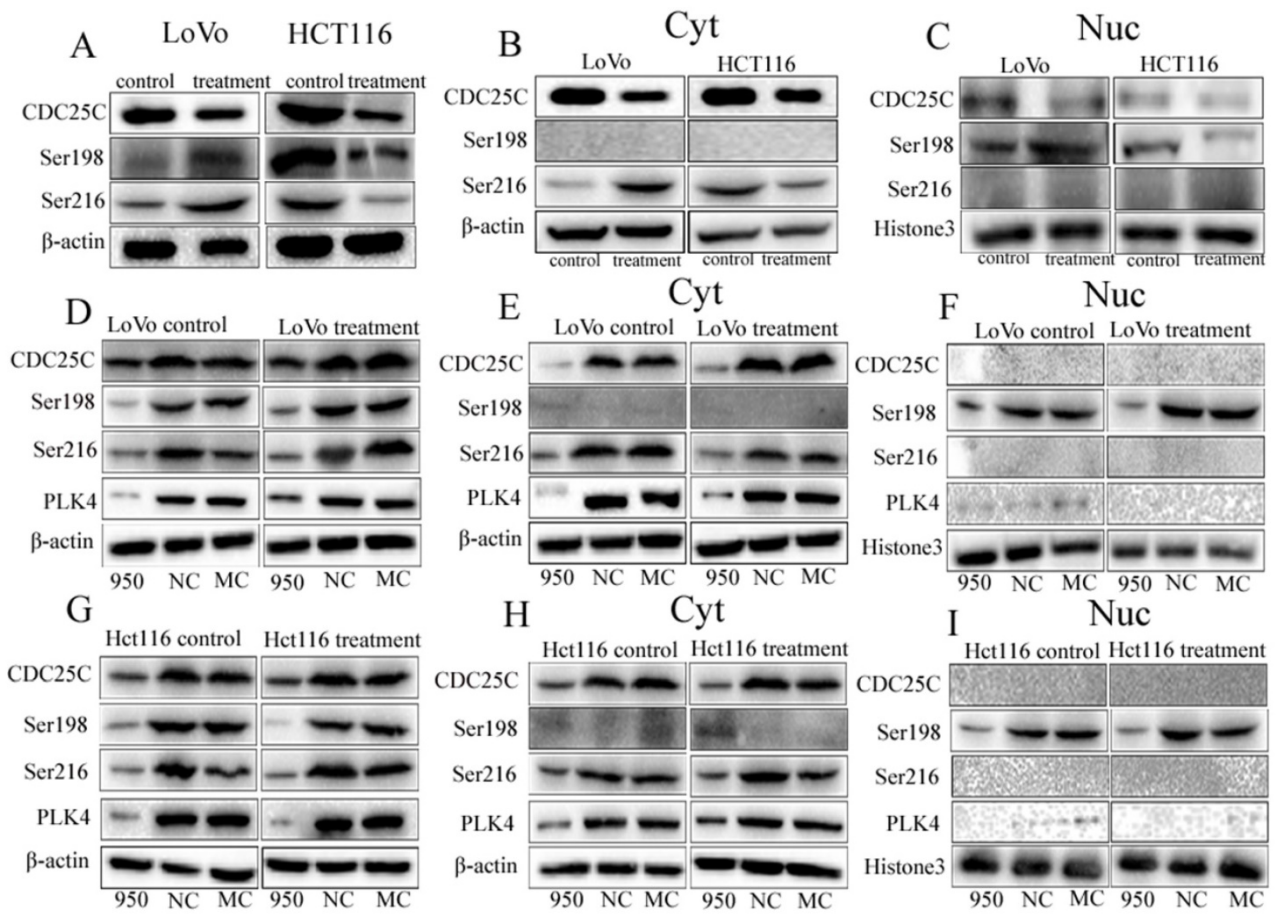
A. WB showing the expression level of CDC25C, pCDC25C-ser198, and pCDC25C-ser216 in LoVo and Hct116 cells treated with and without bufalin. B. WB showing the expression levels of CDC25C, pCDC25C-ser198, and pCDC25C-Ser216 in cytoplasmic from LoVo and Hct116 cells treated with and without bufalin. C. WB showing the expression levels of CDC25C, pCDC25C-ser198, and pCDC25C-ser216 in nuclear from LoVo and Hct116 cells treated with and without bufalin. D. WB showing the total expression levels of PLK4, CDC25C, pCDC25C-ser198, and pCDC25C-ser216 in LoVo cells with PLK4 knocked down. E. WB showing the cytoplasmic expression levels of PLK4, CDC25C, pCDC25C-ser198, and pCDC25C-ser216 in LoVo cells with PLK4 knocked down. F. WB showing the nuclear expression levels of PLK4, CDC25C, pCDC25C-ser198, and pCDC25C-ser216 in LoVo cells with PLK4 knocked down. G. WB showing the total expression levels of PLK4, CDC25C, pCDC25C-ser198, and pCDC25C-ser216 in Hct116 cells with PLK4 knocked down. H. WB showing the cytoplasmic expression levels of PLK4, CDC25C, pCDC25C-ser198, and pCDC25C-ser216 in Hct116 cells with PLK4 knocked down. I. WB showing the nuclear expression levels of PLK4, CDC25C, pCDC25C-ser198, and pCDC25C-ser216 in Hct116 cells with PLK4 knocked down. NC, negative control; MC, mock control; Ser198, pCDC25C Ser198; Ser216, pCDC25C-ser216; Cyt, cytoplasm; Nuc, cell nucleus; WB, western blot analysis.

Results of WB showed that expression of CDC25C was downregulated in both LoVo and Hct116 cells treated with bufalin compared with the control cells (Fig. 6A). The expression of pCDC25C-ser216 and pCDC25C-ser198 was upregulated in LoVo cells treated with bufalin, and decreased in bufalin-treated Hct116 cells compared with the control cells (Fig. 6A). Results of cytoplasmic and nuclear protein separation assays exhibited that cytoplasmic expression of pCDC25C-ser216 were decreased in Hct116 cells treated with bufalin, but elevated in LoVo cells treated with bufalin in comparison to the cells without bufalin treatment (Fig. 6B). Nuclear expression of pCDC25C-ser198 was decreased in Hct116 cells treated with bufalin, but increased in LoVo cells treated with bufalin (Fig. 6C). The differences of CDC25C, pCDC25C-ser216 and pCDC25C-ser198 expression in LoVo and Hct116 cells before and after bufalin treatment were statistically significance (Fig. S3A-abcdefg). Similar results were also confirmed by ICC staining. CDC25C was decreased expression both in cytoplasm and nucleus of LoVo and Hct116 (Fig. S3B). The expression of pCDC25C-ser216 located in the cytoplasm was increased in LoVo PGCCs with daughter cells, and decreased in Hct116 PGCCs with daughter cells compared with the control cells (Fig. S3C). The expression of pCDC25C-ser198 located in the nucleus was increased in LoVo PGCCs with daughter cells but was decreased expression in Hct116 PGCCs with daughter cells (Fig. S3D).

### 11. The expression of CDC25C, pCDC25C-ser216, and pCDC25C-ser198 after PLK4 knockdown

To revealed relationship of PLK4 and CDC25C participating in the formation of PGCCs and their progeny cells, the expression levels of pCDC25C-ser198, pCDC25C-ser216, and CDC25C were detected in LoVo and Hct116 cells upon PLK4 knockdown. As a result, the expression of CDC25C, pCDC25C-ser216 and pCDC25C-ser198 was decreased in LoVo and Hct116 control cells and PGCCs with daughter cells after PLK4 knockdown (Fig. 6D, G). Additionally, results of WB for cytoplasmic and nuclear proteins separation assay confirmed that levels of both CDC25C and pCDC25C-ser216 were decreased in the cytoplasm and the expression of pCDC25C-ser198 was decreased in the cell nucleus after PLK4 knockdown (Fig. 6E-F, H-I). This data showed that PLK4 might be involved in the formation of PGCCs with their progeny cells through regulating the expression of CDC25C and associating with its phosphorylation sites at ser198 and ser216.

### 12. Expression of PLK4 in xenografts and human CRC tissues

**Figure 7:**
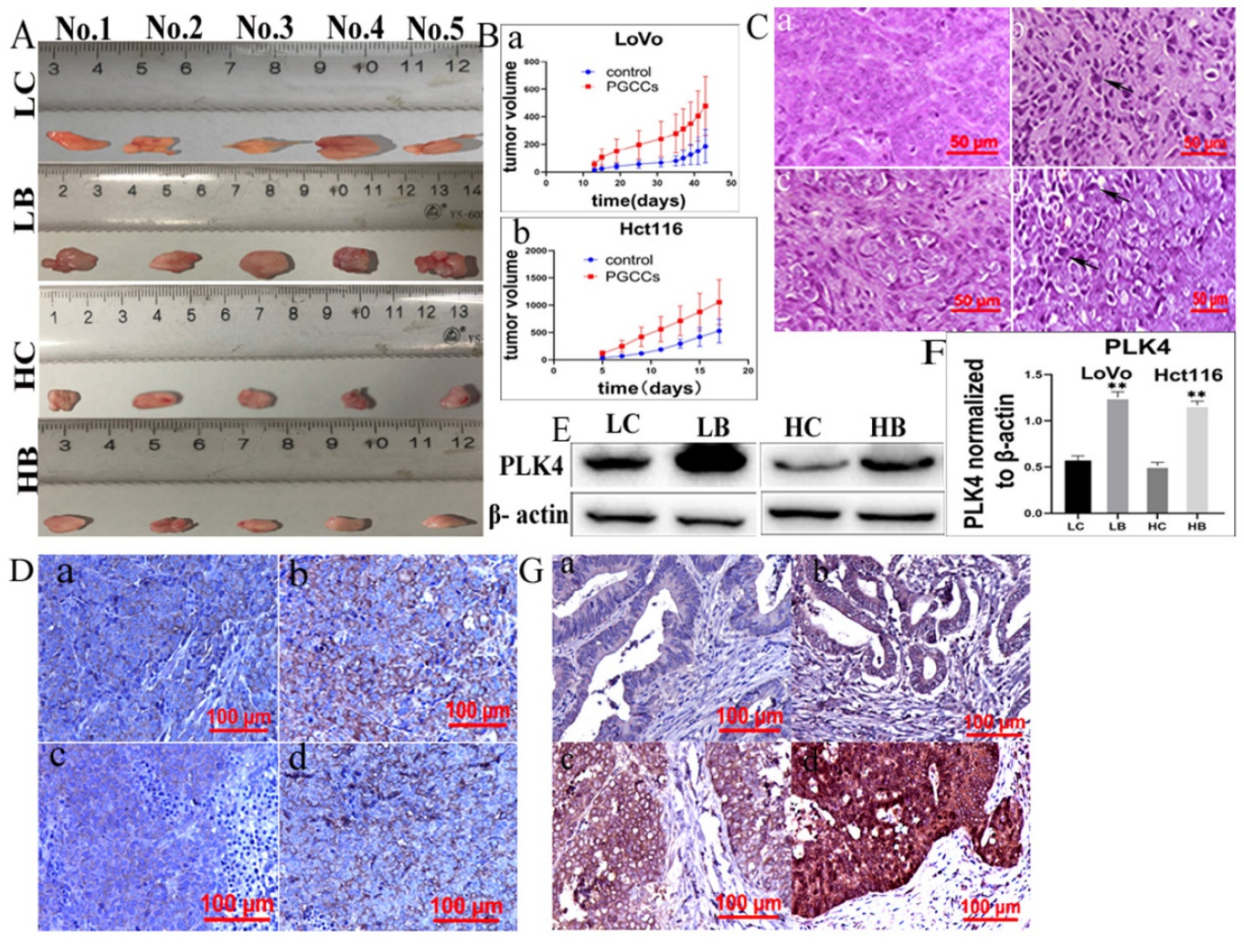
A. pictures showing tumor tissues inoculated with LoVo and Hct116 cells before and after bufalin treatment. B. Growth curves of xenograft-inoculated (a) LoVo cells and (b) Hct116 cells treated with and without bufalin. C. H&E staining of xenografts with and without bufalin treatment (400×). (a) LoVo cells without bufalin treatment, (b) bufalin-treated LoVo cells, (c) Hct116 cells without bufalin treatment, (d) bufalin-treated Hct116 cells. D. IHC of PLK4 in tumor tissues inoculated with LoVo and Hct116 cells before and after bufalin treatment (200×). (a) LoVo cells without bufalin treatment, (b) bufalin-treated LoVo cells, (c) Hct116 cells without bufalin treatment, (d) bufalin-treated Hct116 cells. E. PLK4 expression in tumor tissues inoculated with LoVo and Hct116 cells treated with and without bufalin showed in WB assay. F. Difference in the gray value of PLK4 expression in tumor tissues inoculated with LoVo and Hct116 cells treated with and without bufalin (**P<0.01). G. IHC staining of PLK4 in human CRC tissues (200×). (a) well-differentiated CRC. (b) moderately differentiated CRC. (c) poorly differentiated CRC. (d) lymph node metastasis of CRC patients. LC, LoVo control cells. HC, Hct116 control cells. LB, LoVo cells treated with bufalin. HB, Hct116 cells treated with bufalin. WB, western blot. CRC, colorectal cancer. IHC, immunohistochemical.

We next further analyzed the expression of PLK4 in xenografts. A gross image of the xenografts was present in figure 7A. Tumor growth curves showed that the growth rate of xenograft tumors inoculated with control cells were slower than those of tumors inoculated with bufalin-treated cells, and the differences had statistically significance (Fig. 7Bab, Table 1). Results of H&E staining showed that there were more PGCCs appeared at the xenografts inoculated with LoVo and Hct116 cells treated with bufalin than those inoculated with control cells (Fig. 7C). IHC staining identified that PLK4 was located in cytoplasm and the expression of PLK4 was increased in tumors injected with LoVo and Hct116 PGCCs with progeny cells compared with those injected with control cells (Fig. 7D). Moreover, WB assay also showed that PLK4 expression was higher in tumor tissues injected with bufalin-treated cells compared to those inoculated with control cells (Fig. 7E).

### 13. PLK4 expression in human CRC tissues

The clinic parameters including gender, age, T stage, Lymph node metastasis and Dukes stage from the 158 CRC patients and CRC patients downloaded from TCGA database were compared. The results showed that there was not statistical difference for the comparison of the gender (*P*=0.916), T stage (*P*=0.139), lymph node metastasis (*P*=0.166), Dukes stage (*P*=0.340) between two groups (Table 2). IHC staining was performed to assess the clinicopathological significance of PLK4 in human CRC tissues. 203 paraffin-embedded human CRC tissue samples were used to observe the expression of PLK4 (Fig. 7G). The results displayed that PLK4 expression was higher in group II compared with that in group I (*P=*0.001), and lower in group II than that in group III (*P=*0.002). Furthermore, PLK4 was higher expression in group IV than group III (*P=*0.022). Difference in PLK4 expression between any two groups was statistically significant (Table 3). These results indicated that PLK4 was overexpressed in the xenografts injected with LoVo and Hct116 PGCCs with progeny cells and was closely associated with degree of malignancy of CRCs.

## Discussion

Bioinformatic analysis is essential for the study of genomic and transcriptomic data generated by next-generation sequencing. The application of R software in sequencing data research offers great advantages in clinical bioinformatics 22. Furthermore, TCGA database contains sequencing data of nearly 12,000 human cancer tissues from 33 different cancer types 23. The stemness index based on mRNAsi was independent stem cell index. The higher the mRNAsi score, the stronger the characteristics of the tumor stem cells 4. Based on mRNAsi, eight hub genes were screened out, and *BUB1B*, *CHEK1*,* PLK4* was correlated with the survival time of patients with CRC.

PGCCs had the characteristics of CSC, could be induced by cobalt chloride, triptolide, arsenic trioxide, paclitaxel, oxaliplatin, fluorouracil, and traditional Chinese medicine in many kinds of cancer cell line 7, 24-28. Evidences have revealed that PGCCs with their progeny cells could affect cancer treatment resistance, recurrence, and metastasis 6, 26, 28, 29. We have previously certified that dysregulating expression of cell cycle-related proteins (CDC25C, PLK1, P53, CHEK1, and CHK2) was involved in the formation of PGCCs 7, 30.

Bufalin, also referred to as Chansu, a well-known traditional Chinese herb, is a bioactive polyhydroxy steroid isolated from *Venenum bufonis* 15. Chansu exhibits anti-tumor activity against several cancers 31. A clinical study from 100 patients with liver cancer showed that Chansu could inhibit the proliferation of cancer, protect liver function, improve quality of life and prolong survival time 32. Result of another clinical trial suggested that Chansu had important antineoplastic properties and might effectively improve quality of life for patients with few side effects 32. Wu *et al* 33 and Xie *et al* 34 showed that bufalin induced apoptosis through caspase-3 activation and JNK-dependent pathway in CRCs. However, Sun X, et al. showed that bufalin could induce cancer cell drug resistance in SKOV-3, CAOV-3, and CAVO-4 cells treated with bufalin 35. In the present study, bufalin successfully induced PGCCs formation in CRC cell lines *in vitro*, and the progeny cells showed decreased expression of E-cadherin and increased expression of snail, vimentin, and twist. In addition, progeny cells generated from PGCCs exhibited strong invasion and migration abilities.

PLK4 is a member of the serine/threonine kinase family that plays a crucial role in cell cycle regulation 36. A previous study showed that decreased expression levels of PLK4 preventing the replication of centrioles, leading to mitotic errors, whereas overexpression of PLK4 resulted in rapid amplification of centrioles 37. In a model of human mammary epithelial cells, centrosomal amplification promoted invasion and migration by increasing cytoplasmic elongation, invasion protrusions, and microtubule nucleation 11. Godinho *et al* showed that overexpression of PLK4 promoted cancer cell invasion and migration 38. It has been reported that PLK4 expression involved in the phosphoinositide 3-kinase/threonine-protein kinases signaling pathway, thus increased the EMT-related proteins expression of neuroblastoma cells 39. In addition, PLK4 strengthens the invasion ability of CRC by regulating the Wnt/β-catenin signaling pathway 14, 40. EMT is associated with CSC properties and drug resistance 41. The loss of E-cadherin is associated with an invasion phenotype 40. N-cadherin, snail, and twist have been shown to contribute to EMT. In addition, snail is considered to be an important regulator in the progression of CSC, and cells that undergo EMT are related to acquired CSC features 40. In our study, we showed that the expression of PLK4 was upregulated in LoVo and Hct116 cells after bufalin treatment both in *vitro* and in *vivo*. CDC25C expression was downregulated in LoVo and Hct116 cells after bufalin treatment compared with cells without bufalin treatment. Furthermore, the expression of snail, vimentin, twist, and N-cadherin was downregulated, whereas E-cadherin expression was upregulated after inhibition of PLK4 expression. The abilities of migration and invasion in LoVo and Hct116 cells treated with bufalin also reduced upon PLK4 knockdown. In addition, the expression of PLK4 was associated with pathological grading and lymph node metastasis in human CRCs.

It was reported that p53 gradually accumulated in the nucleus and acted as a transcriptional regulator in response to stress 42. Our research group has previously indicated that different p53 genotypes regulated the expression levels and subcellular location of pCDC25C-ser216 and pCDC25C-ser198, which was associated with the formation of PGCCs 8. CDC25C in the cytoplasm cannot activate the cyclin B1/CDK1 complex, resulting in the arrest of G2/M phase and the formation of PGCCs 8. Phosphorylation of CDC25C at Ser198 by PLK1 and PLK3 promotes to the CDC25C nuclear translocation 13, 43. Ser216 of CDC25C can be phosphorylated by CHEK1 and CHK2 kinases, which promotes to the CDC25C nuclear output, and results in G2/M arrest of the cell cycle 8. Li *et al* 44 suggested that PLK4 expression could be strongly inhibited by wild-type p53 in malignant tumors. Another study showed that p53 suppressed PLK4 expression under stress stimuli 42. PLK4 is a protein kinase, can phosphorylate CDC25C, and PLK4 depletion downregulates the expression of CDC25C and reduces the chance of CDC25C to enter cell nucleus 13, 45.

**Figure 8:**
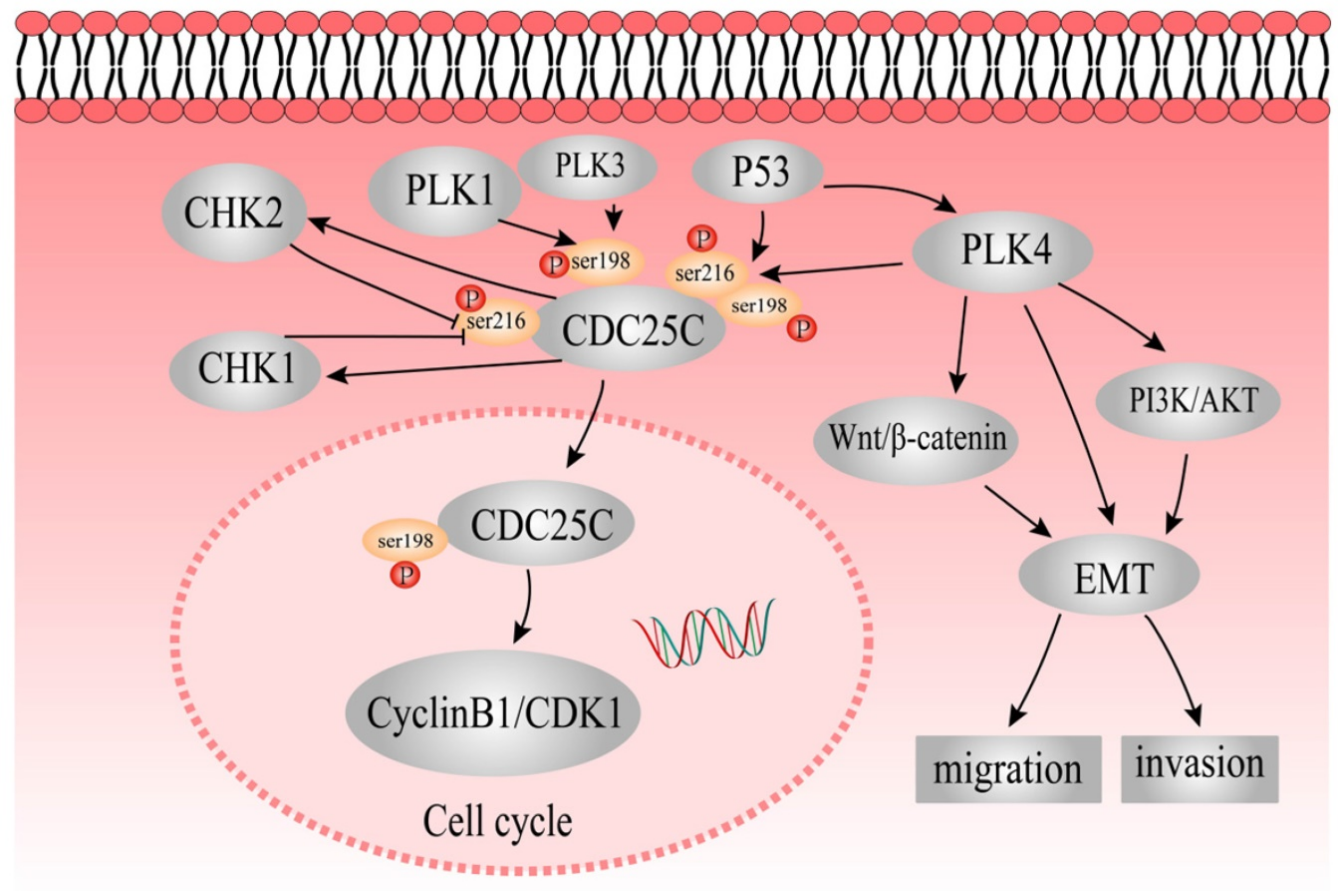
The mechanisms underlying role of PLK4 and CDC25C in the formation of PGCCs and the role of PLK4 in promoting the migration and invasion of PGCCs. P53 acts upstream of CDC25C and PLK4. PLK4 might regulate CDC25C expression by phosphorylating ser198 and ser216 residues. P53 could regulate CDC25C by phosphorylation at ser198 and ser216 residues. pCDC25C-ser198 is located in the cell nucleus, whereas pCDC25C-ser216 is located in the cytoplasm. PLK4 also modulates EMT directly or indirectly through PI3K/Akt and Wnt/β-catenin pathways to promote invasion and migration of PGCCs and progeny cells.

In this study, expression of pCDC25C-ser198 and pCDC25C-ser216 was upregulated in LoVo cells and downregulated in Hct116 cells. The differential expression and subcellular location of pCDC25C-ser198 and pCDC25C-ser216 in the two different cell lines might be due to different *p53* genotypes in LoVo (harboring mutant *p53*) and Hct116 (harboring wild-type *p53*). We also showed that pCDC25C-ser198 levels decreased in the nucleus and the protein cannot be detected in the cytoplasm, whereas pCDC25C-ser216 levels decreased in the cytoplasm and has no expression in the cell nucleus. The expression of pCDC25C-ser216, pCDC25C-ser198, and CDC25C was downregulated in control and PGCCs with progeny cells after inhibition of PLK4 compared with those without inhibition of PLK4, respectively. PLK4 regulates the expression of CDC25C, pCDC25C-ser216 and pCDC25C-ser198, which may be associated the formation of PGCCs. The simple signaling pathway of PLK4 that elucidates its participation in the formation and function of PGCCs is shown in Figure 8. The expression and phosphorylation of CDC25C is regulated by PLK1, PLK3, PLK4, P53, CHEK1, and CHK2. The detailed molecular mechanisms of CDC25C expression and subcellular location of phosphorylated CDC25C before and after bufalin treatment need to be further studied in the future.

## Conclusion

Our results showed that bufalin successfully induced the formation of PGCCs *in vitro*, and the derived progeny cells showed strong migration, invasion, and proliferative abilities. Moreover, PLK4 was overexpressed in cancer cells after bufalin treatment, both in *vivo* and in *vitro*. By mediating the expression of EMT-related proteins, PLK4 regulated the migration, invasion, and proliferative abilities of progeny cells derived from PGCCs. In addition, PLK4 could regulate the expression of pCDC25C-Ser198, pCDC25C-Ser216, and CDC25C associating with the formation of PGCCs. Furthermore, PLK4 was closely associated with the degree of tumor differentiation and lymph node metastasis in human CRC tissues.

## Supplementary Material

Supplementary figures and tables.

## Figures and Tables

**Table 1:** The volume of xenografts injected with LoVo and Hct116 control and PGCCs with daughter cells after bufalin treatment.

Cells	Group	n	Tumor growth (mm^3^)	*P*
LoVo	control cells	5	186.20±121.49	0.028
	PGCCs with daughter cells	5	479.00±212.27	
Hct116	control cells	5	528.60±218.99	0.035
	PGCCs with daughter cells	5	1056.20±410.90	

**Table 2:** The clinic parameters between patients downloaded from TCGA database and enrolled from our institute have been compared.

		TCGA database	Data from our institute	χ^2^	*P*
		n	n		
Gender	Male	226	86	0.011	0.916
	Female	193	72		
Age	≤60	125	77	18.015	0.000
	>60	294	81		
T	T1+T2	88	24	2.194	0.139
	T3+T4	331	131		
Lymph node metastasis	Yes	251	84	1.923	0.166
	No	168	73		
Dukes stage	A+B	243	83	0.912	0.340
	C+D	176	72		

**Table 3:** Comparison of PLK4 staining index among different groups of human CRC tissues.

	group	n	staining index	value of statistics	*P*
Well-differentiated CRCs	I	46	1.20 ± 0.81	χ^2^ = 122.069	0.000*
moderately differentiated CRCs	II	53	2.87 ± 1.90180		
Poorly differentiated CRCs	III	48	4.69 ± 2.21365		
Lymph node metastatic foci	IV	56	6.77 ± 2.57958		

*P*< 0.05: statistically significant. (*P*: difference among the three groups;* P*1 (difference between groups I and II)=0.001; *P*2 (difference between groups II and III)=0.002; *P*3 (difference between groups III and IV)=0.022; *P*4 (difference between groups I and IV)=0.000; *P*5 (difference between groups II and IV)=0.000. CRC, colorectal cancer.

## Abbreviations

PLK: polo like kinase
PGCCs: polyploid giant cancer cells
TTK: threonine tyrosine kinase
CSC: cancer stem cell
CDC25C: cell division cycle 25C
ICC: immunocytochemical
IHC: immunohistochemical
DNA2: DNA replication helicase 2
WB: western blots
siRNA: small interfering RNA
KIF23: kinesin family member 23
DEGs: differently expressed genes
EMT: epithelial-mesenchymal transition
CHK: checkpoint kinase
H&E: hematoxylin and eosin
CRC: colorectal cancer
BUB1B: budding uninhibited by benzimidazoles 1 Homolog Beta
MCM10: minichromosome maintenance 10
BUB1: Budding uninhibited by benzimidazoles 1. WGCNA, weighted gene co-expression network analysis
TCGA: The Cancer Genome Atlas
GS: gene significance
MM: module membership
RT-PCR: real-time PCR
