# Supplementary material for: PLK4 is a key molecule in the formation of PGCCs and promotes invasion and migration of progeny cells derived from PGCCs
Source: J Cancer. 2022 Jul 18;13(9):2954–69. doi: 10.7150/jca.74211 (PMC9330457; doi:10.7150/jca.74211)
Supplement: Supplementary file 1 — Supplementary figures and tables. [file jcav13p2954s1.pdf]

Supplementary figure legends

Supplementary figure1:A. The scatter plot of the three most significant gene modules: (a) black module, (b) yellow module, and (c) blue module. B. The survival curve of the five genes screened using TCGA online tool. a. *MCM10*. b. *TTK*. c. *BUB1*. d. *DNA2*. e. *KIF23*. C. PLK4 expression in ncm460 and LoVo, Hct116. D. Histogram showed the statistical results of PLK4 expression between ncm460 and LoVo, Hct116 (*P < 0.01).

TCGA, the Cancer Genome Atlas.

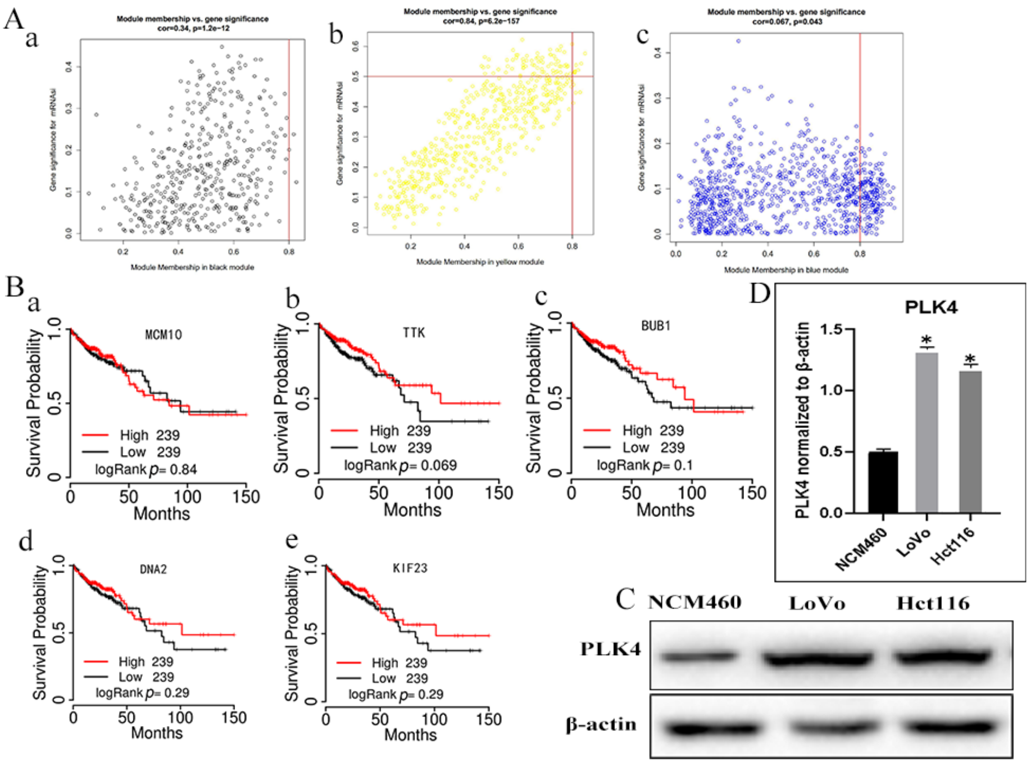

Supplementary figure 2: A. Comparison of the migration rate of bufalin-treated LoVo and Hct116 cells with or without CFI-400945 using Wound-healing assay. B. Comparison of the migration and invasion abilities of bufalin-treated LoVo and Hct116 cellswith or without CFI-400945 using transwell assay (200×). C. Comparison of the number of cell colonies after 30, 60 and 120 bufalin-treated LoVo and Hct116 cells with or without CFI-400945 treatment were incubated in 12-well plates. D. Column diagram of the functional experiment. a. Column diagram of colony formation efficiency (a), migration (b,d) and invasion (c) abilities of LoVo and Hct116

cells treated with bufalin (**$P < 0.01$).

LC, LoVo control cells. HC, Hct116 control cells. LB, LoVo cells treated with bufalin. HB, Hct116 cells treated with bufalin.

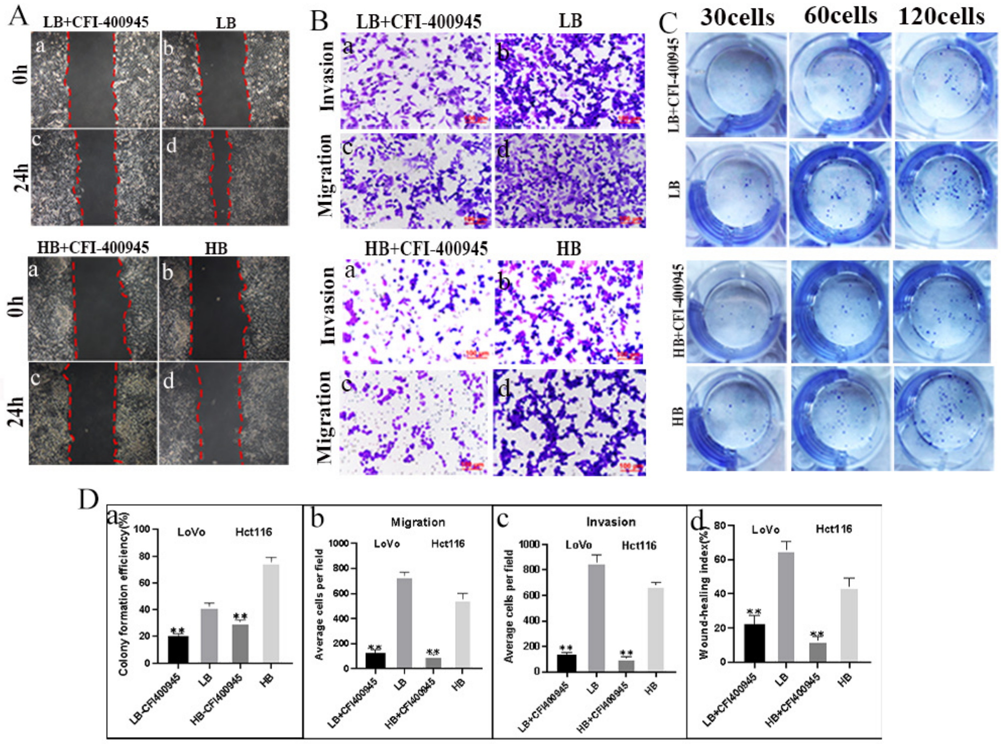

Supplementary figure 3: A. Column diagram showed the differences of CDC25C, pCDC25C-ser198, and pCDC25C-ser216. CDC25C(a), pCDC25C-ser198(b), pCDC25C-ser216(c) expression in total protein in LoVo and Hct116 cells treated with and without bufalin (**$P < 0.01$, *$P < 0.05$). pCDC25C-ser198 expression in cell nucleus (d), CDC25C expression in cytoplasm (e) and nucleus (f) in LoVo and Hct116 cells treated with and without bufalin. pCDC25C-ser216 expression in cytoplasm (g). B. ICC staining of CDC25C in LoVo and Hct116 cells (200×). (a) CDC25C in LoVo cells without bufalin treatment. (b) CDC25C in LoVo cells treated with bufalin. (c) CDC25C in Hct116 cells without bufalin treatment. (d) CDC25C in Hct116 cells treated with bufalin. C. ICC staining of pCDC25C-ser216 in LoVo and Hct116 cells with and without bufalin treatment (200×). (a) pCDC25C-ser216 in LoVo cells without bufalin treatment. (b) pCDC25C-ser216 in LoVo cells after bufalin treatment. (c) pCDC25C-ser216 in Hct116 cells without bufalin treatment. (d) CDC25C-Ser216 in Hct116 cells with bufalin treatment. D. ICC staining of pCDC25C-ser198 in LoVo

and Hct116 cells with and without bufalin treatment (200×). (a) pCDC25C-ser198 in LoVo cells without bufalin treatment. (b) pCDC25C-ser198 in LoVo cells after bufalin treatment. (c) pCDC25C-ser198 in Hct116 cells without bufalin treatment. (d) CDC25C-Ser198 in Hct116 cells with bufalin treatment.

ICC, immunocytochemical.

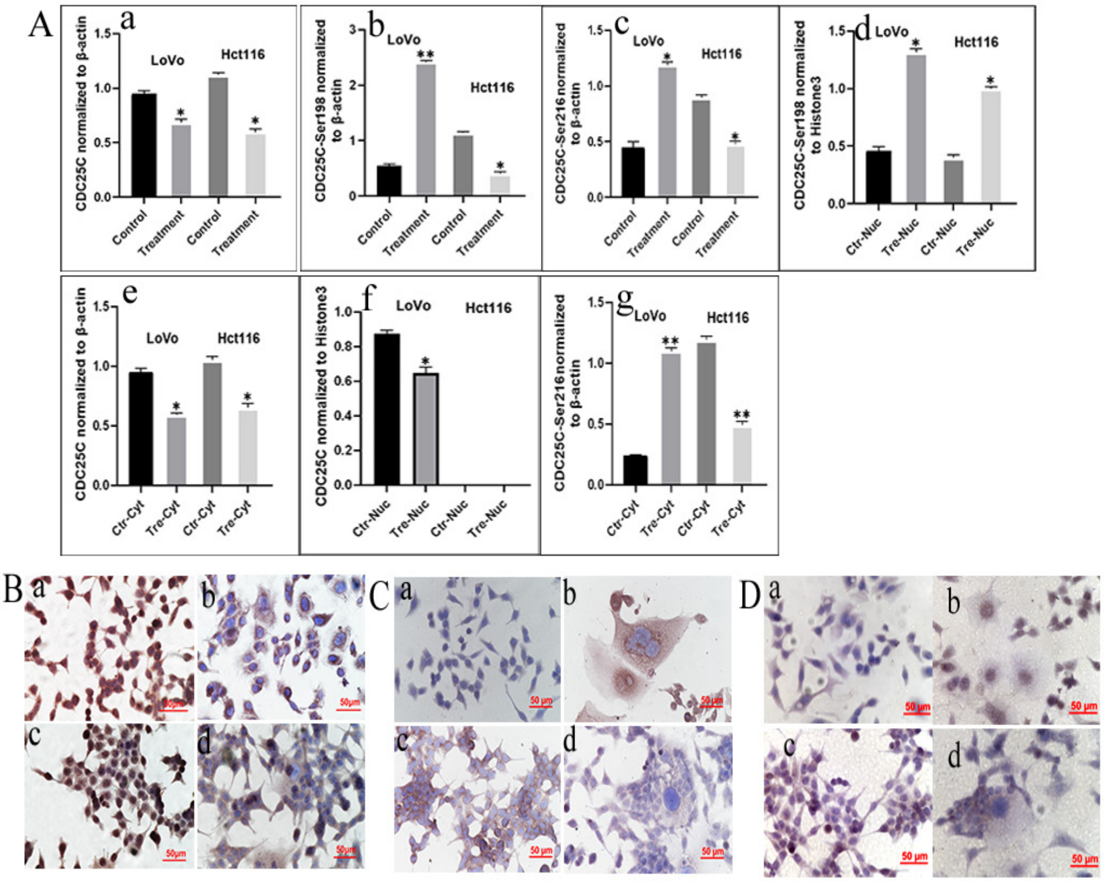

Supplementary table 1. Detailed information of the antibodies utilized in this study.

Antibody	Company	Dilution
E-cadherin	Proteintech	1:50000(WB); 1:500(ICC)
Vimentin	Immunoway	1:2000(WB); 1:200(ICC)
Snail	Novus	1:1000(WB); 1:500(ICC)
Twist	Proteintech	1:1000(WB); 1:500(ICC)
N-cadherin	Wanlei	1:500(WB)
PLK4	Abbkine	1:1000(WB)
PLK4	Affnity	1:1000(WB); 1:400(ICC/IHC)
GAPDH	Zhongshan, Beijing	1:1000(WB)
CDC25C	Abcam	1:2500(WB)
CDC25C	Affnity	1:500(WB);1:300(ICC)
PCDC25C-Ser216	Affnity	1:200(WB); 1:200(ICC)
PCDC25C-Ser198	Affnity	1:200(WB);1:200(ICC)
β-actin	Zhongshan, Beijing	1:1000(WB)
Histone3	Affnity	1:1000(WB)
Anti-rabbit IgG	Cell Signaling Technology	1:3000(WB)
Anti-mouse IgG	Cell Signaling Technology	1:3000(WB)

WB: Western blot；ICC:Immunocytochemistry staining；IHC: Immunohistochemical staining

Supplementary table 2. Sequences of small interfering RNAs (siRNA) targeted to the PLK4.

SiRNA	Sense (5'-3')	Antisense (5'-3')
PLK4i-482	CCUUCUAUCUUGGAGCUUUTT	AAAGCUCCAAGAUAGAAGGTT
PLK4i-950	GCAGAUUAUGAAAUGCCAUTT	AUGGCAUUUCAUAAUCUGCTT
PLK4i-1136	GCCACAAUUUCUACUGCAATT	UUGCAGUAGAAAUUGUGGCTT
PLK4i -PC	CACUCAAGAUUGUCAGCAATT	UUGCUGACAAUCUUGAGUGAG
PLK4i -NC	UUCUCCGAACGUGUCACGUTT	ACGUGACACGUUCGGAGAATT

PC: GAPDH positive control; NC: Negative control, PLK4i: PLK4 knockdown.
